# Case Report: Spontaneous coronary artery dissection presenting as MINOCA in uncontrolled Graves’ disease

**DOI:** 10.3389/fcvm.2026.1885077

**Published:** 2026-07-02

**Authors:** Yunfeng Pan, Hao Han, Jijie Chen, Shiyi Zhang

**Affiliations:** 1 Department of Cardiovascular Medicine, Yiwu Hospital of Traditional Chinese Medicine, Yiwu, China; 2 Department of Endocrinology, Yiwu Hospital of Traditional Chinese Medicine, Yiwu, China

**Keywords:** case report, Graves' disease, MINOCA, spontaneous coronary artery dissection, thyrotoxicosis

## Abstract

**Background:**

Spontaneous coronary artery dissection (SCAD) is an increasingly recognized cause of myocardial infarction with non-obstructive coronary arteries (MINOCA). Although Graves' disease is a well-established cause of coronary vasospasm, its potential association with structural coronary arterial injury remains incompletely understood. We report a case of hyperthyroidism-associated SCAD that provides clinical insight into a possible link between severe thyrotoxicosis and coronary arterial vulnerability.

**Case presentation:**

We report a 56-year-old female with uncontrolled Graves’ disease presenting with an acute ST-segment elevation myocardial infarction (STEMI). Despite prominent inferior ST-segment elevations, emergent coronary angiography revealed a long narrowing extending from the mid to the distal tip of the left anterior descending (LAD) artery with preserved TIMI 3 flow. The lesion's lack of response to intracoronary nitroglycerin supported a working diagnosis of MINOCA. Given the concurrent severe thyrotoxicosis and the substantial risk of precipitating thyroid storm during invasive evaluation, a staged diagnostic approach was adopted: acute-phase management prioritized conservative cardiovascular and antithyroid therapy, while definitive intravascular imaging was deferred. Follow-up intravascular ultrasound (IVUS) at three months demonstrated a persistent intramural hematoma, confirming the diagnosis of SCAD. The patient remained asymptomatic at the one-year follow-up.

**Conclusions:**

This case suggests that severe thyrotoxicosis may contribute to coronary arterial vulnerability and the development of SCAD. It also highlights the diagnostic challenges of complex MINOCA presentations in patients with uncontrolled hyperthyroidism. In selected cases, a staged diagnostic strategy with deferred intracoronary imaging after endocrinological stabilization may provide a safe and effective approach to diagnostic confirmation while minimizing procedural risk.

## 1 Introduction

Spontaneous coronary artery dissection (SCAD) is an increasingly recognized cause of myocardial infarction with non-obstructive coronary arteries (MINOCA) (1). While Graves’ disease is a well-established cause of high-output hemodynamics and coronary vasospasm (2), its potential association with structural coronary arterial injury, including SCAD, remains incompletely understood.

Managing hyperthyroidism-associated MINOCA presents a unique clinical challenge because definitive diagnosis of SCAD often relies on intravascular imaging (3), whereas acute invasive procedures may increase the risk of precipitating thyroid storm in patients with severe thyrotoxicosis (4). Here, we report a case of MINOCA in a patient with uncontrolled Graves’ disease that was ultimately confirmed as SCAD through deferred intravascular imaging. This case provides clinical insight into a possible association between severe thyrotoxicosis and coronary arterial vulnerability. Furthermore, it illustrates how a staged diagnostic approach—prioritizing endocrinological stabilization before delayed intravascular evaluation—may facilitate diagnostic confirmation while minimizing procedural risk.

## 2 Case report

### 2.1 Initial presentation

A 56-year-old female experienced a 3-day history of precordial pain, accompanied by exertional chest tightness and diaphoresis, with episodes lasting approximately 50 min. During an initial evaluation at a local facility shortly after symptom onset, her B-type natriuretic peptide (BNP) was elevated (694.62 ng/L), but high-sensitivity cardiac troponin I (hs-cTnI) and creatine kinase (CK) levels were normal. Due to persistent exertional dyspnea and palpitations, she presented to our institution on day 3 of her illness. She was initially triaged to the respiratory department before being transferred to the cardiology unit. Upon admission, her vital signs included a blood pressure of 142/90 mmHg and a heart rate of 128 beats/min. Physical examination revealed mild bilateral lower extremity edema without jugular venous distension. The lungs were clear to auscultation, and cardiovascular examination demonstrated normal heart sounds without murmurs.

### 2.2 Past medical history

The patient had no prior history of traditional atherosclerotic cardiovascular risk factors, including hypertension, diabetes mellitus, hyperlipidemia, or tobacco use, and reported no recent infections. Although her admission blood pressure was mildly elevated (142/90 mmHg), it normalized during hospitalization and remained within the normal range throughout follow-up without antihypertensive therapy. In addition, 24-h ambulatory blood pressure monitoring was unremarkable, providing no evidence of chronic hypertension. Furthermore, she was postmenopausal and had no history of pregnancy-related vascular complications, hormone replacement therapy, oral contraceptive use, or significant psychosocial stress. There was no family history of premature cardiovascular disease, spontaneous coronary artery dissection, or sudden cardiac death. The patient reported no history or clinical features suggestive of fibromuscular dysplasia (FMD), connective tissue disorders, autoimmune diseases, or migraine disorders.

Two months prior to admission, she was evaluated in an outpatient clinic for bilateral lower extremity edema. Thyroid function testing revealed severe hyperthyroidism: triiodothyronine (T3) 2.64 ng/mL (reference: 0.80–1.90), thyroxine (T4) 21.28 μg/dL (5.00–13.00), free T3 (FT3) 12.9 pmol/L (3.5–7.0), free T4 (FT4) 50.46 pmol/L (10.00–22.00), and fully suppressed thyroid-stimulating hormone (TSH <0.005 μIU/mL). A positive thyrotropin receptor antibody (TRAb) titer of 2.35 IU/L confirmed the diagnosis of Graves’ disease. She received diuretic therapy, which temporarily alleviated the edema, but she did not initiate antithyroid medications due to poor compliance.

### 2.3 Diagnostic evaluation

The initial electrocardiogram (ECG) demonstrated sinus tachycardia with ST-segment elevation in the inferior leads (II, III, aVF) and concurrent ST-T wave abnormalities in the precordial leads (V3–V6) (Figure 1A). Cardiac biomarkers confirmed acute myocardial injury, featuring a peak high-sensitivity cardiac troponin I of 10.916 ng/mL, alongside elevated creatine kinase (497 U/L) and CK-MB (68 U/L). Transthoracic echocardiography (TTE) revealed a preserved left ventricular ejection fraction (58%), impaired diastolic function, and mild mitral regurgitation. Crucially, the absence of regional wall motion abnormalities or apical ballooning effectively rendered Takotsubo syndrome less likely.

**Figure 1:**
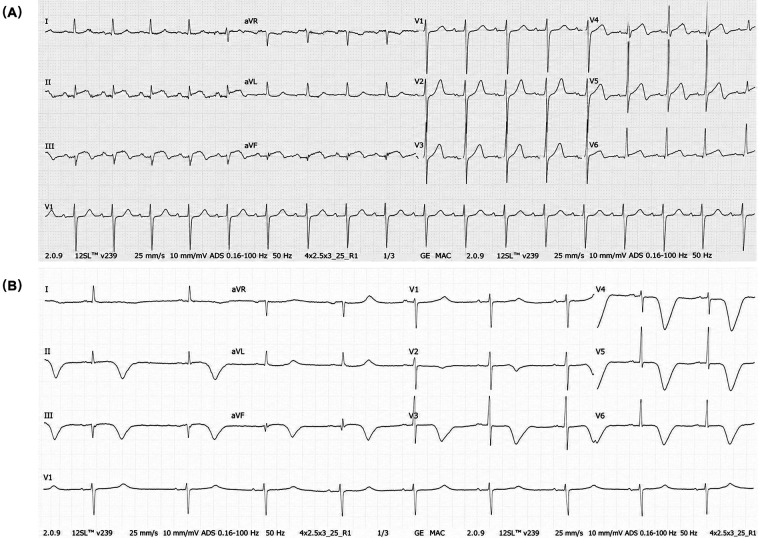
Serial electrocardiogram (ECG) changes. **(A)** The initial ECG on admission demonstrates sinus tachycardia with ST-segment elevation in the inferior leads (II, III, aVF) and concurrent ST-T wave abnormalities in the precordial leads (V3–V6). **(B)** Follow-up ECG prior to discharge exhibits expected evolutionary changes, including T-wave inversion in the inferior and precordial (V3–V6) leads, corresponding to the resolution of clinical symptoms. ECG, electrocardiogram.

Emergent coronary angiography revealed no acute thrombotic occlusion. The left anterior descending (LAD) artery exhibited a 30%–50% long narrowing extending from the mid segment, adjacent to the second diagonal branch, to the distal tip, with preserved Thrombolysis in Myocardial Infarction (TIMI) 3 flow (Figure 2A; Supplementary Video 1). The right coronary artery (RCA) was a small-caliber, non-dominant vessel without significant stenosis (Figure 2B). Intracoronary nitroglycerin administration did not resolve the LAD narrowing, making an isolated vasospastic mechanism unlikely. Consequently, these clinical and angiographic findings fulfilled the initial diagnostic criteria for MINOCA.

**Figure 2:**
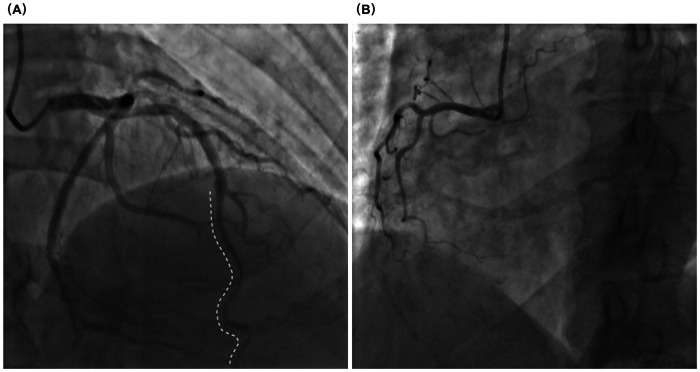
Emergency coronary angiography. **(A)** Angiogram of the left coronary artery demonstrates a 30%–50% long narrowing extending from the mid-LAD, adjacent to the second diagonal branch, to the distal LAD tip (white dashed lines). **(B)** Angiogram of the right coronary artery (RCA) reveals a small-caliber, non-dominant vessel without significant stenosis. LAD, left anterior descending artery; RCA, right coronary artery.

However, this specific angiographic appearance—a long narrowing mimicking a non-obstructive plaque—raised a high clinical suspicion for underlying SCAD. Given the substantial mechanical risk of iatrogenic dissection propagation in such a friable, long lesion involving a tortuous distal LAD, and the prohibitive systemic risk of precipitating a thyroid storm, we deliberately opted for a staged diagnostic approach, deferring definitive intravascular evaluation until endocrinological stabilization. The overarching clinical course, integrating her prior thyroid history, acute cardiovascular presentation, and subsequent long-term follow-up, is visually summarized in Figure 3.

**Figure 3:**
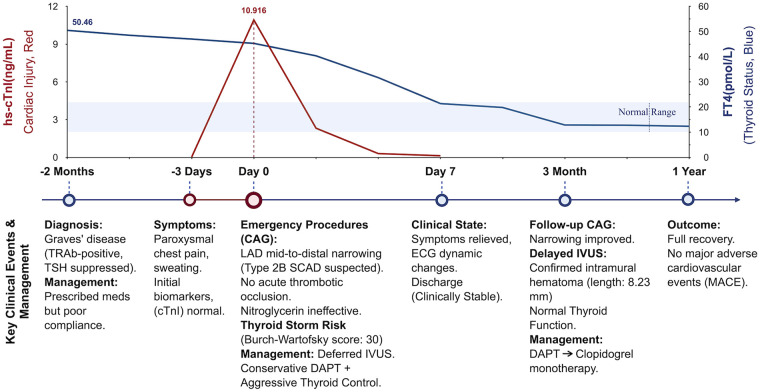
Chronological timeline of clinical events,biomarker trajectories,and patient management. The dual-axis graph illustrates the dynamic changes in high-sensitivity cardiac troponin I (hs-cTnI, red line, left Y-axis) indicative of cardiac injury, and free thyroxine (FT4, blue line, right *Y*-axis) reflecting thyroid status, from 2 months prior to admission through the 1-year follow-up. The light blue horizontal shaded band across the graph denotes the normal reference range for FT4 (10.00–22.00 pmol/L), visually illustrating the patient's transition from a severe thyrotoxic state to euthyroidism. Key clinical milestones—including the emergent coronary angiography for suspected spontaneous coronary artery dissection (SCAD), the deferral of intravascular imaging due to impending thyroid storm, and the subsequent confirmation of an intramural hematoma via delayed IVUS—are annotated sequentially along the horizontal time axis. CAG, coronary angiography; DAPT, dual antiplatelet therapy; IVUS, intravascular ultrasound; MACE, major adverse cardiovascular events; TRAb, thyrotropin receptor antibody; TSH, thyroid-stimulating hormone.

### 2.4 Management and follow-up

Initially, the patient received standard acute coronary syndrome pharmacotherapy, including dual antiplatelet therapy (DAPT; aspirin and ticagrelor) and a high-intensity statin. Following angiography, given the preserved TIMI 3 flow and the suspicion of SCAD, we established a strictly conservative cardiovascular strategy. Crucially, her severe thyrotoxicosis was accompanied by a Burch-Wartofsky Point Score (BWPS) score (5) of 30, suggesting an impending thyroid storm. In keeping with the staged approach, treatment of thyrotoxicosis was prioritized before further invasive assessment. She was promptly treated with propylthiouracil to suppress thyroid hormone synthesis and propranolol for strict rate control.

During hospitalization, her clinical symptoms resolved, serial ECGs showed expected evolutionary changes, including T-wave inversion in the inferior and precordial leads (Figure 1B), and cardiac biomarkers progressively normalized.

At the 3-month follow-up, laboratory tests confirmed the complete normalization of her thyroid profile. Follow-up coronary angiography demonstrated partial angiographic improvement of the previously noted long narrowing in the LAD (Figure 4A; Supplementary Video 2). Concurrent deferred intravascular ultrasound (IVUS) confirmed the persistence of an 8.23-mm-long intramural hematoma lacking an intimal tear in the mid-to-distal segment (Figures 4B–D,F; Supplementary Video 3). Additionally, a distinct, non-obstructive plaque (plaque burden: 47%) was incidentally noted in the mid-LAD proximal to the second diagonal branch (Figure 4E), which was independent of the culprit SCAD lesion. Following the IVUS confirmation of SCAD and sustained clinical stability, her DAPT regimen was de-escalated to clopidogrel monotherapy to minimize long-term bleeding risk and alleviate gastrointestinal intolerance. During subsequent follow-ups at 6 and 12 months, the patient remained asymptomatic. Follow-up echocardiography demonstrated preserved left ventricular systolic and diastolic function, and serial ECGs remained stable without recurrent ischemic changes. Thyroid function remained within the euthyroid range on a tapering dose of propylthiouracil. Notably, propranolol was successfully discontinued at 3 months following resolution of severe thyrotoxicosis.

**Figure 4:**
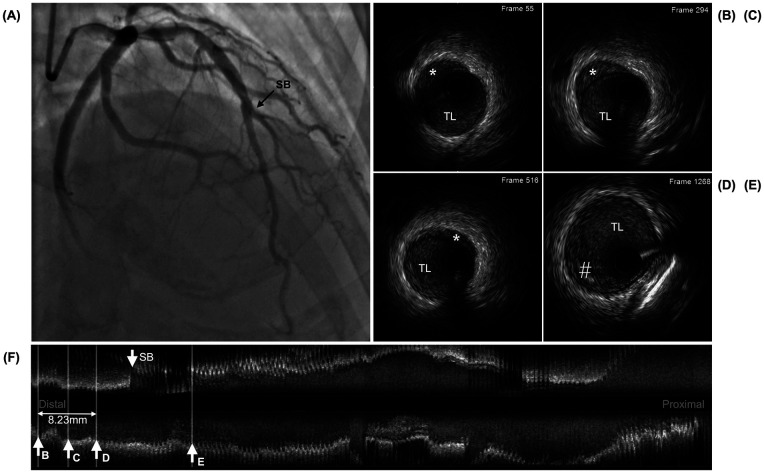
Coronary angiography and intravascular ultrasound (IVUS) imaging at 3-month follow-up. **(A)** Follow-up angiogram of the left coronary artery demonstrates partial improvement of the previously noted long narrowing in the left anterior descending (LAD) artery; the black arrow indicates the second diagonal branch (SB). **(B–D)** Cross-sectional IVUS images demonstrating an intramural hematoma (*) alongside the patent true lumen (TL). **(E)** Cross-sectional IVUS image showing a plaque (**#**) in the mid-LAD segment, proximal to the second diagonal branch. **(F)** Longitudinal IVUS reconstruction of the interrogated vessel segment. The corresponding locations of the cross-sectional images **(B–E)** are indicated by upward white arrows labeled B, C, D, and E, respectively. The double-headed arrow delineates the longitudinal extent of the intramural hematoma, measuring 8.23 mm. The downward white arrow denotes the location of the side branch (SB). IVUS, intravascular ultrasound; LAD, left anterior descending artery; SB, side branch; TL, true lumen.

## 3 Discussion

SCAD is an increasingly recognized cause of MINOCA, predominantly affecting middle-aged women (6). In acute settings, it frequently eludes detection due to the absence of a classic radiolucent intimal flap or double-lumen appearance (7). In the present case, emergent angiography demonstrated a long segment of diffuse narrowing extending from the mid-LAD to the distal vessel. In the absence of an identifiable intimal tear and with preserved TIMI 3 flow, the lesion was initially approached within the diagnostic framework of MINOCA. However, the characteristic long, diffuse stenotic pattern extending to the distal vessel tip angiographically classified the lesion as Type 2B SCAD according to Saw's criteria (7). Subsequent deferred IVUS definitively confirmed the underlying pathology as an intramural hematoma, establishing the final diagnosis.

This case highlights the diagnostic challenges encountered in complex MINOCA presentations, particularly when electrocardiographic findings appear discordant with the angiographic anatomy. The inferior STEMI pattern accompanied by precordial ST-T abnormalities initially appeared disproportionate to the isolated long narrowing of the LAD. In addition, severe thyrotoxicosis is a recognized trigger for Takotsubo syndrome, which may closely mimic acute coronary syndromes in this clinical setting (8). Although preserved LVEF and the absence of apical ballooning on echocardiography made typical Takotsubo syndrome less likely, alternative MINOCA etiologies, including myocarditis, coronary thromboembolism, plaque disruption, and type 2 myocardial infarction, were also systematically considered. However, the characteristic long tapering LAD lesion, absence of an embolic source, and subsequent IVUS demonstration of an intramural hematoma collectively favored SCAD, although myocarditis could not be completely excluded initially without cardiac magnetic resonance (CMR). These diagnostic uncertainties still warranted definitive intracoronary imaging. Although low-contrast or near-zero-contrast IVUS is technically feasible (9, 10), acute IVUS was intentionally deferred in this case. The decision reflected concern about iatrogenic dissection propagation in a long Type 2B lesion involving a tortuous, small-caliber distal LAD with preserved TIMI 3 flow, where the anticipated diagnostic benefit was outweighed by the procedural risk. This mechanical concern was further compounded by the systemic risk of precipitating a procedure-related thyroid storm during the acute thyrotoxic phase. Accordingly, a staged diagnostic strategy was adopted, with IVUS deferred until thyroid function had been adequately stabilized. This approach allowed definitive confirmation of the underlying intramural hematoma while minimizing both procedural and systemic risks.

The coexistence of uncontrolled Graves’ disease and SCAD in this patient suggests a potential link between severe thyrotoxicosis and coronary arterial vulnerability. Excess thyroid hormones and autoimmune activity trigger an inflammatory cascade, wherein cytokines (e.g., IL-6 and TNF-α) exacerbate oxidative stress (11). This environment upregulates matrix metalloproteinases (MMP-7 and MMP-9), degrading collagen and elastin in the tunica media and compromising structural integrity (12, 13). Compounding this endothelial dysfunction, uncontrolled hyperthyroidism superimposes severe hemodynamic stress. Specifically, tachycardia, increased cardiac output, and catecholamine surges amplify mechanical shear stress against the vulnerable intima, ultimately precipitating dissection (14).

A potential interaction between SCAD and coronary artery spasm (CAS) should also be considered. Although an isolated vasospastic mechanism was considered unlikely given the lack of angiographic response to intracoronary nitroglycerin, transient severe vasospasm may still have contributed to the development of SCAD. In a structurally vulnerable coronary artery, intense vasoconstriction may generate sufficient mechanical stress to disrupt the vasa vasorum or precipitate an intimal tear, thereby providing a plausible link between transient vasomotor dysfunction and structural arterial injury (3, 15).

Management of hyperthyroidism-associated SCAD requires a rigorous, multidisciplinary strategy. Conservative cardiovascular management remains the cornerstone for hemodynamically stable patients, as unnecessary percutaneous coronary intervention (PCI) may increase the risk of iatrogenic dissection propagation in highly fragile vessels (16). Equally important is prompt control of the underlying thyrotoxic state. Restoration of euthyroidism may reduce both hemodynamic stress and ongoing vascular injury, thereby creating a more favorable environment for spontaneous arterial healing.

Antiplatelet therapy in conservatively managed SCAD remains challenging because clinicians must balance ischemic protection against the potential risks of hematoma expansion and bleeding (17). Although antiplatelet monotherapy may theoretically reduce the risk of intramural hematoma expansion, treatment decisions in the acute phase must also account for the initial clinical presentation. In this case, the patient presented with STEMI, while the diagnosis of SCAD remained presumptive because definitive intravascular imaging had been intentionally deferred. Given the potential consequences of undertreating an acute coronary syndrome before the underlying mechanism was fully established, a more cautious strategy was adopted. Accordingly, dual antiplatelet therapy (aspirin and ticagrelor) was initiated as a temporary treatment strategy to maintain clinical stability until both the diagnosis and vascular healing could be more definitively assessed. Once clinical stability was achieved and partial angiographic healing was documented at 3 months, treatment was individualized by de-escalating to single antiplatelet therapy (SAPT) to reduce long-term bleeding risk. Delayed IVUS provided objective confirmation of both the SCAD etiology and the persistent intramural hematoma, supporting subsequent de-escalation of antiplatelet therapy. In addition, although high-intensity statin therapy was initially prescribed in accordance with contemporary STEMI management, follow-up reassessment identified an incidental 47% plaque burden in the mid-LAD on IVUS. This finding supported continued statin therapy for secondary atherosclerotic prevention, while the intensity was appropriately reduced from high- to moderate-intensity therapy during the convalescent phase to improve long-term tolerability.

Follow-up IVUS was safely performed three months after presentation, by which time thyroid function had substantially improved. To minimize the risk of mechanical injury to the small-caliber distal vessel, the IVUS catheter was not advanced to the extreme distal tip of the lesion. Despite this conservative imaging approach, interrogation of the proximal portion of the lesion demonstrated a persistent 8.23-mm intramural hematoma, providing further confirmation of the SCAD diagnosis and indicating that incomplete hematoma resorption remained present at three months.

Because acute intravascular imaging had been intentionally deferred, follow-up invasive angiography with IVUS was selected at 3 months to confirm the underlying diagnosis rather than relying solely on coronary computed tomography angiography (CCTA). Although CCTA has an established role in the follow-up of SCAD, its diagnostic performance may be limited in small-caliber or tortuous mid-to-distal coronary segments, such as the Type 2B lesion observed in this case (18). In addition, noncalcified atherosclerotic plaques may occasionally mimic intramural hematomas on CCTA, further reducing diagnostic confidence (19). Given the importance of excluding persistent structural abnormalities and guiding subsequent antiplatelet de-escalation, a more definitive anatomical assessment was considered necessary. Because follow-up angiography had already been planned to assess luminal recovery, concurrent IVUS provided additional anatomical information while ensuring imaging within the true lumen, which was subsequently confirmed by visualization of an intact and patent vessel lumen.

Although the intentional deferral of acute intravascular imaging precluded direct assessment of the initial hematoma burden, partial angiographic improvement of the LAD lesion together with sustained clinical and electrocardiographic stability strongly suggests progressive healing rather than ongoing disease activity. The persistence of a residual intramural hematoma at three months is also consistent with the known healing course of SCAD. Previous studies have shown that spontaneous anatomical healing of SCAD is often gradual and may require 3–6 months or longer to achieve complete resolution (20). Accordingly, residual abnormalities identified during early follow-up should be interpreted cautiously, as incomplete healing does not necessarily indicate persistent disease activity. Although restoration of euthyroidism likely reduces ongoing hemodynamic stress, recovery of the affected arterial segment may still require a prolonged period of vascular remodeling. These observations support an individualized follow-up strategy in patients with hyperthyroidism-associated SCAD, particularly when confirmation of structural healing may influence subsequent management decisions.

Several limitations of this report should be acknowledged. The intentional deferral of acute-phase intracoronary imaging and provocative testing precluded real-time evaluation of the culprit lesion and vasomotor reactivity. However, this diagnostic compromise was clinically mandated to mitigate the prohibitive risk of iatrogenic dissection propagation and a procedural thyroid storm. In addition, CMR imaging was not performed because severe thyrotoxicosis, extreme resting tachycardia, and concern regarding impending thyroid storm rendered prolonged imaging assessment impractical during the acute phase. Consequently, comprehensive characterization of myocardial injury and exclusion of focal myocarditis were limited. Lastly, the proposed mechanistic link between thyrotoxicosis and coronary arterial fragility inherently relies on clinical deduction, as direct *in vivo* histological validation is unattainable in this setting.

## 4 Conclusion

In this case, uncontrolled Graves' disease was associated with SCAD and may have contributed to coronary arterial vulnerability through a combination of inflammatory, autoimmune, and hemodynamic mechanisms. The case also highlights the diagnostic challenges of complex MINOCA presentations when acute intravascular imaging is not feasible. In selected patients with severe thyrotoxicosis, a staged diagnostic strategy with deferred intracoronary imaging after endocrinological stabilization may provide a safe and effective means of confirming the diagnosis while minimizing procedural risk. Close collaboration between cardiovascular and endocrine teams remains important to optimize both vascular recovery and thyroid disease control.

## 5 Patient perspective

“I was initially terrified when I was told I was having a heart attack, especially since I had always considered myself relatively healthy aside from some recent weight loss and palpitations. Learning that my untreated thyroid condition might have been related to my heart problem was surprising. I am grateful for the careful, step-by-step treatment approach, which avoided unnecessary invasive procedures during a period of severe illness. Over the past year, my symptoms have completely resolved with medication, and I have fully returned to my normal daily activities.”

## Data Availability

The original contributions presented in the study are included in the article/Supplementary Materials, further inquiries can be directed to the corresponding author.

## Supplementary material

Supplementary Video 1 Baseline coronary angiography. The video demonstrates a long, diffuse narrowing extending from the mid-segment to the distal tip of the left anterior descending (LAD) artery. This characteristic angiographic pattern is consistent with Type 2B spontaneous coronary artery dissection (SCAD). Thrombolysis in Myocardial Infarction (TIMI) 3 flow is preserved throughout the vessel.

Supplementary Video 2 Three-month follow-up coronary angiography. The video demonstrates partial angiographic improvement of the long narrowing in the mid-to-distal left anterior descending (LAD) artery, consistent with the gradual anatomical healing of the dissected segment.

Supplementary Video 3 Intravascular ultrasound (IVUS) pullback at three-month follow-up. The pullback in the left anterior descending (LAD) artery reveals a persistent intramural hematoma alongside the true lumen, definitively confirming the diagnosis of spontaneous coronary artery dissection (SCAD). Additionally, an incidental 47% atherosclerotic plaque burden is visible in the mid-LAD proximal to the SCAD lesion. To minimize the risk of mechanical injury to the small-caliber distal vessel, the IVUS catheter was deliberately not advanced to the extreme distal tip of the lesion.
